# Examination of School Children’s Teeth

**Published:** 1902-03-15

**Authors:** 


### EXAMINATION OF SCHOOL CHILDREN'S TEETH.

The National Dental Association, through the Seventh District Association of New York State, recently presented a request to the Board of Education, through Dr. Boyd G. Saunders. It is desired that permission be given for the inspection of the teeth of the school children of Rochester—or at least an inspection of a sufficient number of grades and schools so that a fair average of the city can be obtained and statistics compiled to be used in connection with similar statistics from other cities throughout the United States.

The movement is a general one, not only in this country, but throughout Europe, and the effort is made to compile figures that will be of scientific value, on which remedies may be based of general application.

Dr. Saunders, who appeared to make the request on behalf of the dental society, said the plan was purely philanthropic and scientific; not one penny of expense would be asked nor any demand made later for appropriations. He did not desire to inspect the teeth of all the children in the schools, but to take certain grades and certain schools that might be regarded as a fair average of the entire city. The work, he said, would be done in a thoroughly scientific manner; the instruments were to be carefully sterilized; very little time would be required for the examination of each pupil, not more than a minute or two.

He said the State Association was anxious to have the work completed as soon as possible; that all the schools in Brooklyn had just been inspected, and the work now reported completed.

This inspection has been completed in Germany and the work was undertaken in the United States as the result of a resolution adopted at the last annual convention of the National Dental Association, held at Old Point Comfort, Va., when a special committee of five was appointed to consider the expediency of inaugurating steps, looking to the co-operation of the public schools in teaching "Good Teeth—Good Health." This committee was directed to report back to the next annual convention.

The work undertaken by the committee was to gather statistics regarding the frequency of dental "caries" and other abnormal conditions of the mouths of school children in the United States. No accurate statistics of this kind, respecting the school children of this country, are available, and reliable information can only be obtained through detailed examinations and reports. It is claimed many points of scientific value can be settled by a systematic examination of the teeth of the school children.

In a circular sent out by the National Dental Association to the local association, an illustration is given of the results obtained of the careful examination of the mouths of about 20,000 children in Germany, of ages ranging from 6 to 15 years, 95 percent of whom showed dental "caries."

Of the whole number of children examined, 372 anomalies of different characters were found, including harelip, cleft palate, irregularities, V-shaped jaw and the like.

These are extracts of the tables compiled in Germany: Number examined, 6 to 8 years, 6,060; 9 to 10 years, 3,518; 12 to 15 years, 5,157. With perfect teeth, 6 to 8 years, 407, or 3.8 percent; 9 to 10 years, 268, or 3.4 percent; 12 to 15 years, 172, or 5.5 percent. Percent with caries: 6 to 8 years, 93 percent; 9 to 10 years, 96.6 percent; 12 to 15 years, 94.5 percent.

The circular of the National Association continues: "We want children instructed in the care of the teeth and mouth; taught some system of oral hygiene. Young minds are very susceptible and they would readily understand, if properly taught, the serious results liable to follow the neglect of their mouthes and teeth. Statistics tabulated from a very large number of examinations can not fail to show most interesting results of scientific value, to which we hope you will promptly contribute your share.

"If, in the furtherance of the work, you will arrange to have made a systematic examination of the teeth of the children in the primary and grammar schools of your section, whose ages range from 6 to 15 years, a sufficient number of examination blanks and diagrams will be sent at the expense of the National Association."

The members of the Board of Education seemed to appreciate fully the value of the work undertaken by the National Dental Association, but they appeared reluctant to grant a formal permit to insect the schools. Commissioner Chamberlain moved the request be granted, but the motion was not seconded. Commissioner Forbes then moved that the matter lay over until the next board meeting for further investigation, when the permit will probably be granted, unless serious objection is made in some quarters.—*Rochester Herald.*

